# Characterization and Evaluation of the Artemis Camera for Fluorescence-Guided Cancer Surgery

**DOI:** 10.1007/s11307-014-0799-z

**Published:** 2014-10-25

**Authors:** P. B. A. A. van Driel, M. van de Giessen, M. C. Boonstra, T. J. A. Snoeks, S. Keereweer, S. Oliveira, C. J. H. van de Velde, B. P. F. Lelieveldt, A. L. Vahrmeijer, C. W. G. M. Löwik, J. Dijkstra

**Affiliations:** 1 Department of Radiology and Molecular Imaging, Leiden University Medical Center, Leiden, The Netherlands; 2 Department of Image Processing, Leiden University Medical Center, Leiden, The Netherlands; 3 Department of Surgery, Leiden University Medical Center, Leiden, The Netherlands; 4 Department of Otorhinolaryngology & Head and Neck Surgery, Erasmus Medical Center, Rotterdam, The Netherlands; 5 Department of Biology, Utrecht University, Utrecht, The Netherlands; 6 Department of Pathology, University Medical Center Utrecht, Utrecht, The Netherlands

**Keywords:** Fluorescence-guided, Near-infrared fluorescence, Optical imaging, Artemis, Camera, Surgery

## Abstract

**Purpose:**

Near-infrared (NIR) fluorescence imaging can provide the surgeon with real-time visualization of, *e.g.*, tumor margins and lymph nodes. We describe and evaluate the Artemis, a novel, handheld NIR fluorescence camera.

**Procedures:**

We evaluated minimal detectable cell numbers (FaDu-luc2, 7D12-IRDye 800CW), preclinical intraoperative detection of sentinel lymph nodes (SLN) using indocyanine green (ICG), and of orthotopic tongue tumors using 7D12-800CW. Results were compared with the Pearl imager. Clinically, three patients with liver metastases were imaged using ICG.

**Results:**

Minimum detectable cell counts for Artemis and Pearl were 2 × 10^5^ and 4 × 10^4^ cells, respectively. *In vivo*, seven SLNs were detected in four mice with both cameras. Orthotopic OSC-19-luc2-cGFP tongue tumors were clearly identifiable, and a minimum FaDu-luc2 tumor size of 1 mm^3^ could be identified. Six human malignant lesions were identified during three liver surgery procedures.

**Conclusions:**

Based on this study, the Artemis system has demonstrated its utility in fluorescence-guided cancer surgery.

**Electronic supplementary material:**

The online version of this article (doi:10.1007/s11307-014-0799-z) contains supplementary material, which is available to authorized users.

## Introduction

In surgery, many non-invasive imaging modalities, such as computed tomography (CT), magnetic resonance imaging (MRI), single-photon emission computed tomography (SPECT), and positron emission tomography (PET), are used in a preoperative setting for the detection of tumors and for surgical planning. Translating these techniques to the operating room is challenging due to altered body positions and tissue manipulation. Therefore, the surgeon still mainly relies on visual inspection and tactile information during surgery. New intraoperative imaging modalities that support the surgeon in identifying vital structures and discriminating healthy from diseased tissues in real-time are needed, which is especially important for laparoscopic procedures where the surgeon lacks tactile information.

Near-infrared (NIR) fluorescence-guided surgery (FGS) is such a novel technique [1, 2]. Compared to SPECT or PET, NIR fluorescence provides high-resolution images, can visualize microscopically tumor nodules, and can be tumor-specific due to targeted exogenous agents [3]. NIR light has the advantage of increased depth penetration and decreased autofluorescence compared to visible light [4, 5]. Furthermore, NIR light is invisible to the human eye and consequently does not alter the surgical field.

The success of FGS in recognizing tumors and vital structures depends to a large extent on the imaging system used. In an excellent review, Gioux *et al.* [6] systematically described the required criteria to which a new clinically applicable NIR fluorescence camera system has to comply. These requirements are translated into a set of practical criteria.

The most important criteria for practical application are the following: field of view, imaging distance to the patient, maneuverability, simultaneous imaging of near-infrared and visible light, real-time imaging, light intensity, sterility, and electrical safety. These criteria mainly affect the design choices of the following camera components: sensor, lens system, light source, and filters/dichroic mirrors.

**Fig. 1:**
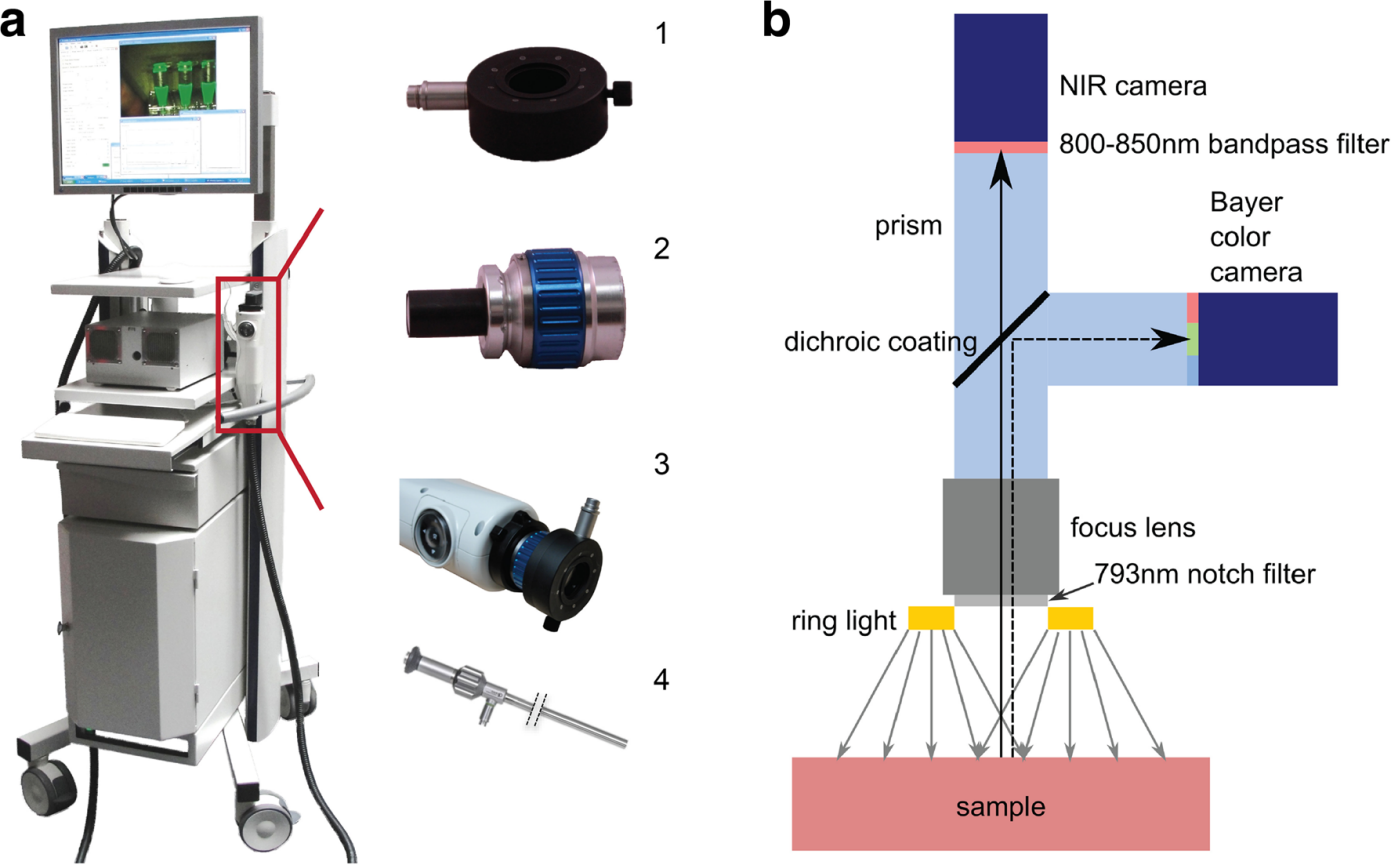
Artemis NIR imaging system. **a** The NIR fluorescence imaging Artemis handheld system is positioned on a movable trolley. Ring light (*1*) and lens (*2*) have to be attached to the handheld camera (*3*) to obtain NIR fluorescence images. Instead of lens and ring light, a scope (*4*) can be attached to the handheld camera when minimal invasive surgery is applied. **b** Schematic representation of the Artemis camera with light path and filters. The sample is illuminated by a ring light around the camera lens.

Currently, a small number of camera systems that fit most of the criteria above are clinically available [7]. The intraoperative Artemis imaging system is recently developed within the Center for Translational Molecular Medicine (CTMM) consortium. The system is developed in close collaboration with the clinic, which resulted in an easily maneuverable system (Fig. 1a) that acquires (NIR) fluorescence and white light images simultaneously allowing for a depicted overlay. Furthermore, the Artemis has an option to assemble a laparoscope to the camera head, allowing for minimally invasive surgery.

The goal of this work was to evaluate the Artemis camera in two oncological procedures in which real-time NIR fluorescence could be of added value: (a) radical tumor resection and (b) the detection of sentinel lymph nodes, the first draining nodes from the tumor. Irradical tumor resections are a major problem in cancer surgery. At present, although tumors clinically appear to be radically resected, high percentages of microscopically irradical resections have been reported at pathological analysis [8, 9]. Such patients require adjuvant treatment and have higher risk of tumor recurrence [10, 11]. When performed with an exogenous tumor-specific ligand, NIR FGS enables intraoperative guidance of tumor resections. This potentially decreases the relatively high percentages of irradical tumor resections and locoregional recurrences, which may lead to increased survival rates and decreased morbidity [8, 9].

The detection of the SLN is of vital importance for cancer staging and consequently influences the choice of therapy and therefore the survival rates. In breast cancer and melanoma surgery, the SLN procedure is presently the standard of care [12]. Currently, two exogenous contrast agents are clinically available: indocyanine green (ICG) (800 nm) and methylene blue (700 nm). Both non-specific contrast agents are used for the visualization of SLNs, vital structures, and various tumors in the clinic [12].

In this study, we performed a preclinical assessment of the sensitivity and intraoperative utility of the Artemis in the detection of head-and-neck tumors and SLNs preclinically in xenograft mouse models.

Images were simultaneously acquired with the Pearl Impulse Small Animal Imager (LI-COR), an existing commercially available and commonly used imaging system. The Pearl system is expected to be an order of magnitude more sensitive than the Artemis, and therefore, these images serve as a ground truth comparison. The Pearl camera does not allow real-time imaging. The sample is shed from outside light imaging in a closed box. Although benefitting the image quality, the latter two characteristics prevent application of the Pearl for intraoperative (pre-)clinical imaging. The Artemis imaging system allows real-time imaging and free access to the sample, but imaging takes place at less ideal circumstances than in the Pearl.

We report the first in-human study performed with the Artemis imaging system where colorectal liver metastases were visualized using ICG.

## Methods

### Near-Infrared Camera Systems

**Fig. 2:**
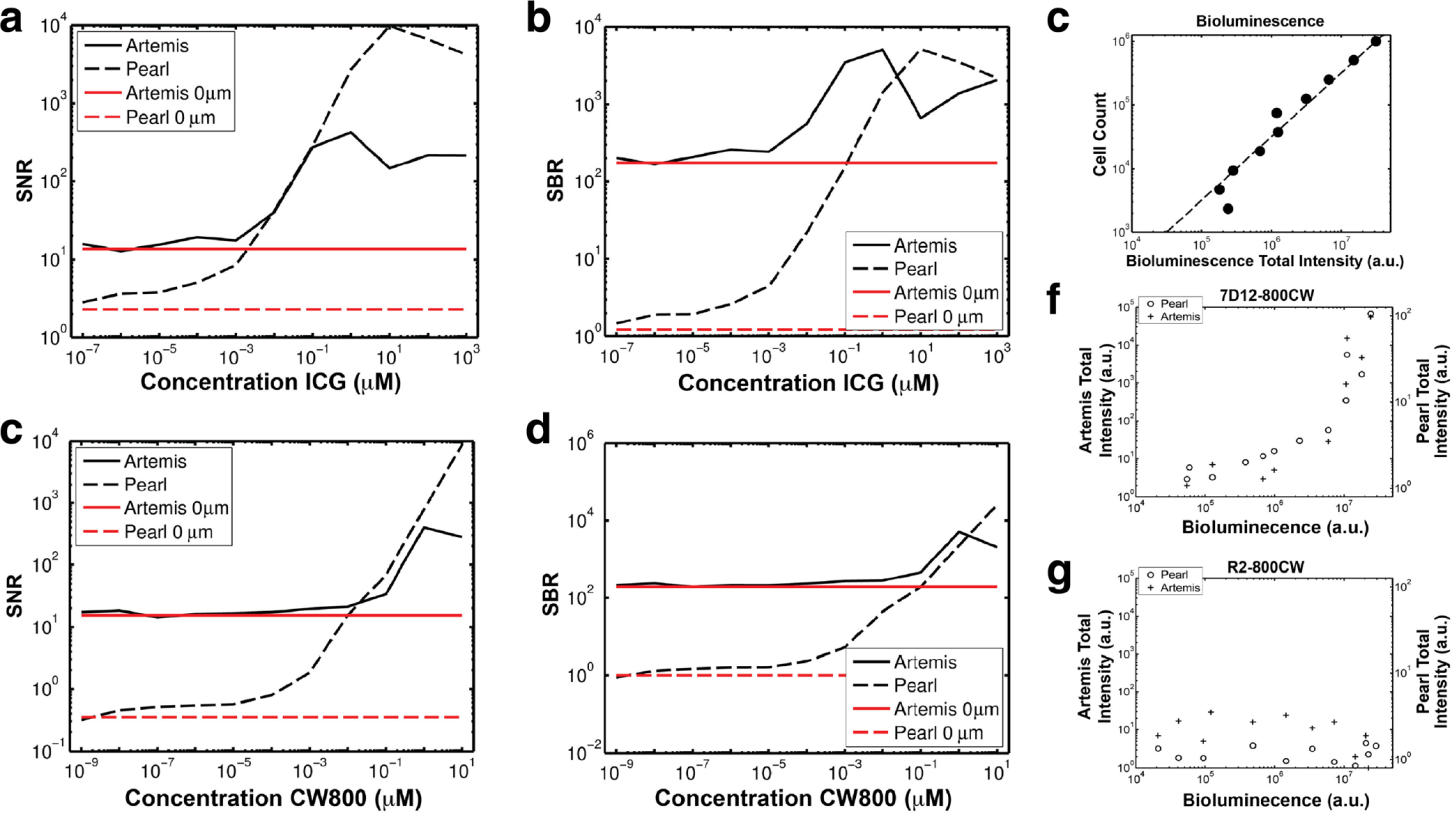
Calibration of the Artemis system and minimal detection limit of hypopharyngeal tumor cells. **a**, **c** Signal-to-noise (*SNR*) and **b**, **d** signal-to-background (*SBR*) ratios of concentration ranges of **a**, **b** ICG and **c**, **d** CW800 imaged in 96-well plates. Measurements from control wells with 0 μM ICG and CW800 are shown as *red horizontal lines*. **e** Bioluminescence was correlated to the number of FaDu-luc2 cells. **f** FaDu-luc2 cells were incubated with the EGFR-specific nanobody 7D12-800CW and **g** non-specific nanobody R2-800CW for 2 hours. After incubation, cells were washed twice and cell pellets containing different amount of cells were imaged using the Artemis and Pearl imaging system.

The Artemis camera system was developed by Quest Medical Imaging and the Leiden University Medical Center (Fig. 1). Images were acquired using custom-designed cameras in a portable, freely moveable camera head. A wide field lens for open surgery was used. Samples were illuminated from a ring containing optical fibers (Fig. 1a-1) attached to the lens (Fig. 1a-2) during open surgery imaging.

A Lumencor light engine was used containing four solid-state light sources for visible light illumination with peak intensities in the blue, cyan, green, and red. For NIR fluorescence imaging, an NIR laser with a peak intensity at 785 nm was used for the preclinical and at 793 nm for the clinical system. The intensities of the light sources could be controlled from the Artemis software. A sterilizable optical fiber was used to connect the light engine to the illumination ring.

Reflected light was captured in the camera head as depicted in Fig. 1b. Reflected excitation light is blocked by a 750–800-nm notch filter. Subsequently, the light passes through a lens that could be used for focusing. The light then enters a prism containing a dichroic coating (<785 mm) in order to separate visible and NIR light. The visible light passes through a low-pass filter (<640 nm) and the NIR light through a high-pass filter (>808 nm). Both light beams are captured by a Sony ICX618 sensor with Bayer configuration having a 640 × 494 pixel grid.

Exposure times and sensor gains were separately adjusted for both imaging channels, and acquisition was synchronized to the longest exposure time. The raw data of both sensors could be saved as individual snapshots or as a real-time movie. During procedures, the visible light channel, the NIR fluorescence channel, and an adjustable overlay are presented.

The Pearl Impulse uses two lasers for excitation with a wavelength of 685 and 785 nm. In this work, only the 785-nm excitation light is used. The Pearl camera automatically optimizes exposure times. Imaging data is acquired with a thermoelectrically cooled CCD sensor.

### Near-Infrared Probe(s)

The Artemis was evaluated in two imaging procedures in which fluorescence-guided surgery could be of added value. The clinically available ICG (Pulsion Medical Systems, Munich, Germany, *λ*_ex_ = 780 nm, λ_em_ = 820 nm) is frequently used in SLN mapping and for that the choice of dye in this study. IRDye 800CW (LI-COR, Lincoln, NE, USA, *λ*_ex_ = 774 nm, λ_em_ = 789 nm) was chosen because it is one of two novel fluorophores in the process of clinical translation [2]. Two imaging procedures were evaluated because of the differences in fluorophores and mainly because fluorophore concentration differs at the side of interest. The near-tumoral injected ICG is highly concentrated compared to the intravenously injected IRDye 800CW conjugated to a targeting moiety. Furthermore, ICG and IRDye 800CW differ in excitation and emission spectra and have different quantum yields.

ICG was resuspended in Cealb (20 % human serum albumin, Sanquin, Amsterdam, The Netherlands) to obtain a dilution from 1 mM to 100 fM. Clinically, 25 mg ICG was resuspended in 10 ml of sterile water before injection obtaining a stock solution of 2.5 mg/ml (3.2 mM). Of this, 4 ml, corresponding to a dose of 10 mg, was administered intravenously.

IRDye 800CW carboxylate was resuspended in phosphate-buffered saline to obtain a dilution from 1 μM to 100 fM. Tumor-specific imaging experiments were done using the epidermal growth factor receptor-specific nanobody 7D12 with the non-epidermal growth factor receptor (EGFR)-specific nanobody R2 as a control [13, 14]. The generation of the nanobodies 7D12 and R2 and the conjugation to the NIR fluorophore IRDye 800CW were done as described previously [15–17].

### Cell Lines

Two human cancer cell lines were used: FaDu-luc2 (human hypopharyngeal squamous cell carcinoma) and OSC-19-luc2-cGFP (metastatic oral squamous cell carcinoma). Both were cultured as previously described [1, 14].

### Camera Characterization *In Vitro*

#### Calibration of Camera System

Concentration series of both NIR fluorophores were used to estimate the concentration-dependent sensitivity.

ICG was dissolved in human serum albumin (HSA) in concentrations from 1 mM to 100 fM. HSA without ICG served as a control. IRDye 800CW was dissolved in PBS in concentrations of 1 μM to 100 fM. PBS without IRDye 800CW served as a control.

One hundred microliters of each concentration was added to a 96-well plate and experiments were performed in duplicate. All series were imaged using both Pearl and the Artemis (with exposure times of 60 ms, to ensure real-time imaging).

#### Cell Experiments

FaDu-luc2 cells were cultured in T75 culture flasks until subconfluence. After washing with binding medium (MEM supplemented with 25 mM Hepes and 1 % BSA, at pH 7.2), 20 ml of binding medium with 50 nM 7D12-800CW was added. Cells were incubated in the dark, for 2 hours in a humidified incubator at 37 °C and 5 % CO_2_. Cells were harvested with a solution of 10 % trypsin in PBS. Subsequently, cells were washed in medium and adjusted to a suspension containing 2 × 10^6^ cells. This suspension was diluted ten times in a 1:2 ratio in medium and aliquoted in 500-μl tubes. Tubes were centrifuged with 13,000 rates per minute, and after the aspiration of medium, cell pellets were imaged with the Pearl and Artemis camera system. After imaging, cells were resuspended in 50 μl of PBS containing 2 μl of d-luciferin solution (Syncem, Inc. Elk Grove Village, IL) followed by bioluminescence imaging (BLI) using the IVIS Spectrum imaging system (Caliper Life Sciences). Quantification of the BLI signal was performed through standardized regions of interest using Living Image software (Caliper Life Sciences). The experiment was performed in duplicate and cells incubated with medium served as a negative control [18]. The same dilutions of cells were made without incubation of 7D12-800CW to correlate the BLI signal to the number of cells (Fig. 2e).

### Camera Characterization *In Vivo*

#### Animal Models

Animal experiments were performed in female nude Balb/c mice (Charles River laboratories, l’Arbresle, France) aged 4–6 weeks. Mice were housed in individually ventilated cages and provided with food and sterilized water *ad libitum*. During the experiments, general health was monitored by weight measurements and tongue inspections. Imaging procedures were performed under isoflurane gas anesthesia (5 % induction and 2 % maintenance). The local animal welfare committee of the Leiden University Medical Center approved the animal experiments.

In order to induce subcutaneous xenografts of hypopharyngeal squamous cell carcinomas, mice were inoculated at four spots on the back with 1, 2, 3, and 4 × 10^6^ FaDu-luc2 cells, diluted in 50 μl PBS. Tumor growth was monitored twice a week with BLI. At day 10, mice were randomly allocated to injection with 7D12-800CW or R2-800CW.

Orthotopic tongue tumors were induced in the tip of the tongue through a submucosal injection of 40,000 OSC-19-luc2-cGFP cells, diluted in 10 μl phosphate-buffered saline. Twice a week, mice body weight was monitored, tongues were inspected, and BLI was measured. At day 20, mice were randomly allocated to injection of 7D12-800CW or R2-800CW. BLI signals served as a control for the tumor specificity of the probe.

#### Sentinel Lymph Node Detection Using ICG

Precontrast images were taken with the Artemis and Pearl imaging systems to obtain background signal intensities of the tissue of interest. The Artemis was configured to image in real time (exposure time of 40 ms). After positioning of the mice (*n* = 4), 10 μl of 100 μM ICG and HSA was injected submucosally in the tip of the tongue. NIR fluorescence imaging of ICG was performed at 0, 5, 10, 15, and 20 min after injection with both the Pearl and Artemis imaging systems. After 20 min, the skin of the cervical region was removed and images were taken. Subsequently, cervical lymph nodes were removed under NIR fluorescence guidance of the Artemis imaging system.

#### Tumor-Specific Imaging Using 7D12-800CW

FaDu-luc2 mice were randomly allocated to the intravenous injection of 7D12-800CW (3.2 nmol, *n* = 4) or R2-800CW (3.0 nmol, *n* = 3). After an incubation of 24 h, imaging was performed using both imaging systems. Tumors of mice injected with 7D12-800CW were excised with direct guidance of the real-time fluorescence signal of the Artemis (exposure time 60 ms). All excised tumors were imaged *ex vivo* with both imaging systems, and tumor volume was determined by measuring the width (*W*), length (*L*), and height (*H*) of each tumor using a digital caliper. Tumor volume was calculated by using the ellipsoid volume formula *π* / 6 × *L* × *W* × *H* [19]. Four tumors of mice injected with 7D12-800CW were cut in half and subsequently divided into halves until submillimeter tumor parts were obtained after which NIR fluorescence images were acquired. Muscle tissue was used as a control.

When OSC-19-luc2-cGFP tumors were visible by the human eye and BLI signal ranged between 5 × 10^9^ and 1 × 10^10^ relative light units (RLU), 7D12-800CW (3.2 nmol, *n* = 3) or R2-800CW (3.0 nmol, *n* = 3) was intravenously injected. Whole body fluorescence imaging with the Pearl and Artemis was performed after 24 h of incubation. Subsequently, all tongue tumors were resected under direct fluorescence guidance of the Artemis camera system.

### Histology and Fluorescence Microscopy

The resected hypopharyngeal squamous cell carcinomas and tongue tumors were cut in two, one half was snap frozen in isopentane and stored at −80 °C. The other half was fixed in formalin overnight and embedded in paraffin. Frozen or paraffin tissue sections of 10μm were air-dried, and fluorescence imaging was performed using the Odyssey (LI-COR) to confirm tumor specificity of 7D12-800CW. Histologic sections were stained with standard hematoxylin–eosin stain (HE). The presence of OSC-19-luc2-cGFP and FaDu-luc2 cells was confirmed by staining the sections with anti-human wide spectrum cytokeratin staining (Abcam Inc., Cambridge, MA, USA).

### Human Liver Metastases

Three patients with suspected colorectal liver metastases, based on a preoperative four-phase CT scan (Aquilion 64; Toshiba, Tokyo, Japan) of the thorax and abdomen, who were planned to undergo surgery with curative intent, were included. Exclusion criteria were pregnancy, lactation, or an allergy to iodine, shellfish, or ICG. Patients received 10 mg of ICG, diluted in 4 ml sterile water, as an intravenous bolus at 24 h prior to surgery. After exploration, the liver was first visually inspected and palpated then intraoperative ultrasound imaging was performed to locate the liver metastases. Subsequently, all liver segments were imaged using the Artemis imaging system. Patients were provided with informed consent, and the study was approved by the Local Medical Ethics Committee of the Leiden University Medical Center, Leiden, The Netherlands and was performed in accordance with the ethical standards of the Helsinki Declaration of 1975.

### Statistical Analysis

All acquired images were analyzed by annotating regions of interest (ROI). During *in vitro* acquisitions, the background ROI was positioned at a location of homogeneous intensity without tissue. For the *in vivo* acquisitions, one background ROI was taken on the animal, next to the structures of interest. Furthermore, a ROI was drawn on a dark area outside the animal or sample for camera background correction.

In each acquisition, mean foreground *μ*_f_ and background *μ*_b_ signals were measured within the ROIs. The camera noise *σ*_*n*_ was estimated as the standard deviation with the annotated homogeneous areas.

Each acquisition is characterized by three measures:

1. Signal-to-noise ratio (SNR): *μ*_f_/*σ*_n_. This indicates how well signals of a particular intensity can be detected.
2. Contrast-to-noise ratio (CNR): (*μ*_f_ − *μ*_b_)/*σ*_n_. This indicates how well different regions can be identified.
3. Signal-to-background ratio (SBR): *μ*_f_/*μ*_b_. This measure is often reported in the literature to evaluate (tumor) marker specificity.

In this work, CNR is mainly used to evaluate the Artemis camera system. This is different than the SBR used in many probe-binding studies. While SBR is essentially a measure for the uptake of a probe, it does not measure how well a camera system is capable of capturing the contrast between tissues with different probe uptake. For the latter, it is essential to take the camera noise into account. A tumor with a high SBR may be almost invisible when imaged with a camera with high noise levels, while a very low SBR may be discernible very well when camera noise is low. Measurements with minimal CNR = 2 were considered reliable. This corresponds to a limit of detection (LOD) defined as the control or background intensity plus two times the noise level.

The SNR, CNR, and SBR for Pearl and Artemis were subjected to a Wilcoxon rank-sum test or *U*-test for non-normally distributed data.

## Results

### Camera Characterization *In Vitro*

#### Calibration of Camera System

Acquisitions of 96-well plates with concentration series of ICG and 800CW were compared between the Artemis and Pearl camera systems based on SNR (Fig. 2a, c) and SBR (Fig. 2b, d). CNR is in this experiment very similar to SNR. The SNR and SBR curves for ICG from the Pearl show an increase in SNR and SBR for increasing concentrations up to 10 μM. After this, peak quenching takes place, causing a decrease in intensity. The SNR and SBR for the Artemis peak earlier. This is, however, due to sensor saturation (*i.e.*, values of 255, the high end of the dynamic range) for the fixed exposure time of 60 ms. For shorter exposure times, higher concentrations could be imaged within the dynamic range. For the lower concentrations, the SNR and SBR curves flatten off at a higher concentration than for the Pearl, indicating 10^−2^ μM as the lower boundary for reliable ICG detection. The SBR for low concentrations of ICG for the Artemis is about 100 instead of 1. This is due to bright reflections of the excitation light in the well plates that penetrates through the emission filter. These reflections were visible in wells with low concentrations of fluorophore as well as the control well and showed the eight bright spots that corresponded to the fiber ends in the illumination ring. The plot in Fig. 2a shows that within the dynamic range of the Artemis at 60 ms exposure time, SNRs are comparable between Artemis and Pearl. *P*-values for the *U*-test were *P* = 0.75 and *P* = 0.09, respectively.

Figure 2c, d shows similar results for the 800CW concentration series as for the ICG concentration series. Bright reflections of the excitation light again cause high SNRs and SBRs for low 800CW concentrations, while the sensor was saturated for 10 μM of 800CW. The minimum detectable concentration of 800CW is again 10^−2^ μM. However, as for the ICG, the excitation light reflections may prevent detection of lower concentrations. *P*-values for the *U*-test were *P* = 0.04 (SNR) and *P* = 0.01 (SBR). These significant differences can be attributed to both a smaller dynamic range of the Artemis, as well as a higher minimally detectable concentration.

#### Cell Line Experiments

Flow cytometry showed EGFR expression of FaDu-luc2 cells (data not shown). The experiment to determine the minimal detection limit of FaDu-luc2 cells was performed using the EGFR-specific nanobody 7D12-800CW and the non-EGFR-specific R2-800CW as a control. The total intensities of fluorescence measured for 7D12-800CW show agreement between Artemis and Pearl (Fig. 2f). Also, although clearly not a linear relationship, both cameras measure an increased amount of total fluorescence for a larger number of cells, as indicated by a higher bioluminescent signal. For the non-specific R2-800CW, both cameras show no relation between bioluminescence and total intensity (Fig. 2g). The Artemis total intensities were higher for the R2-800CW experiment than for the low bioluminescent cell pellets with 7D12-800CW, while Pearl total intensities are comparable. We attribute this (small) variation to variations in sample placement under the Artemis. The minimum amount of cells that could be detected with the Artemis was extrapolated from the bioluminescence signal (Fig. 2e) and proved to be approximately 2 × 10^5^ cells and 4 × 10^4^ cells using the Artemis and Pearl, respectively.

### Camera Characterization *In Vivo*

#### Sentinel Lymph Node Detection Using ICG

**Fig. 3:**
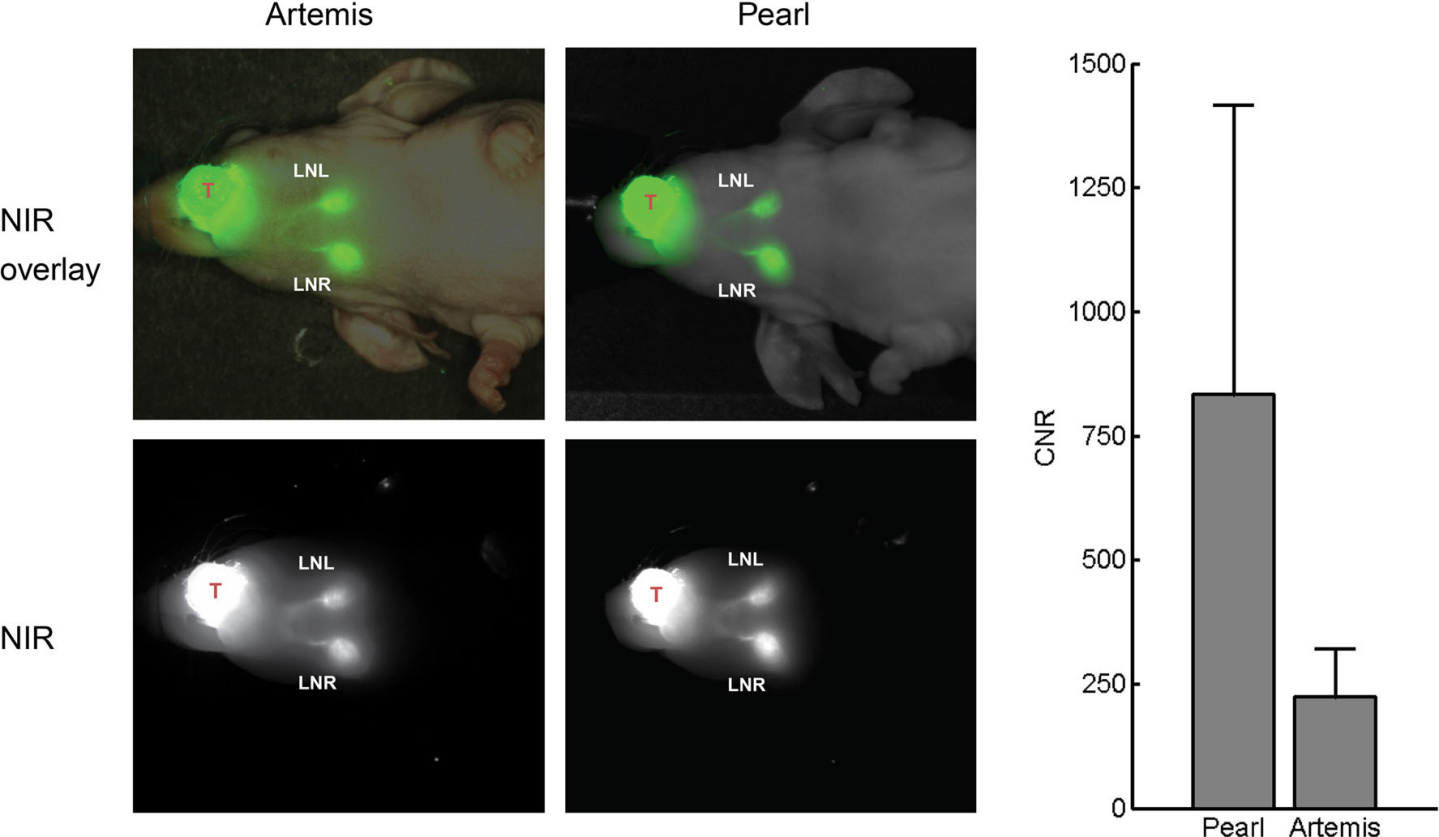
Sentinel lymph node detection using indocyanine green. Cervical sentinel lymph nodes could clearly be identified after injection of 100 μM ICG (10 μl) in the tongue (*T*) using both the Artemis and Pearl imaging system within 10 min after injection. Contrast-to-noise ratios (CNR) of the Artemis and Pearl are shown. *LNL* = lymph node left; *LNR* = lymph node right.

Using both the Artemis and Pearl camera, seven SLNs were detected *in vivo* in four mice. All lymph nodes were visible within 10 min after injection. Massaging the injection spot could have expedited this process. After removal of the skin, all eight lymph nodes were visible. The initially invisible lymph node was covered by strongly absorbing tissue and was visible after removal of skin. Figure 3 shows NIR fluorescence images acquired with Artemis and Pearl after ICG injection in the tongue, as well as overlays with reflectance images. The distribution of ICG in the lymph node, as well as the lymphatic ducts, is clearly visible using both camera systems. CNRs are computed for each lymph node, where the background ROI is positioned between the front paws of the mouse. The mean CNR (Pearl 833, Artemis 225) and standard deviations (Pearl 584, Artemis 96) for the seven detected lymph nodes with closed skin are shown in Fig. 3. Signal-to-background ratios were 126 (standard deviation (SD) 59) for the Pearl and 1,260 (SD 691) for the Artemis. This difference was mainly due to low background signals, where the Pearl background was relatively higher. SBR is an unreliable measure when the background signal is low. The *U*-test *P*-values showed significant differences for both CNR (*P* = 0.010) and SBR (*P* < 0.001).

#### Tumor-Specific Imaging Using 7D12-800CW, Tongue Tumor Model

**Fig. 4:**
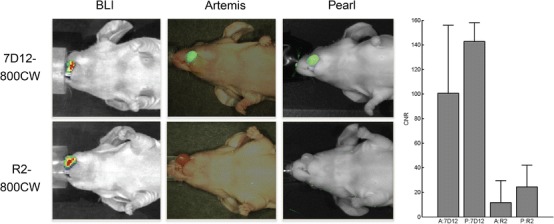
Near-infrared fluorescence delineation of orthotopic tongue tumors. OSC-19-luc2-cGFP tongue tumors could clearly be identified after injection of the epidermal growth factor receptor-specific nanobody 7D12-800CW (50 μg) using both the Artemis and Pearl. No fluorescence could be observed after injection of 50 μg of control nanobody R2-800CW. Contrast-to-noise ratios (*CNR*) calculated by using the Artemis and Pearl are shown. *A* = Artemis; *P* = Pearl.

Orthotopic EGFR-overexpressing (data not shown) OSC-19-luc2-cGFP tongue tumors were clearly identifiable using both Artemis and Pearl after injection of 7D12-800CW (Fig. 4). Bioluminescence confirmed the location of the tumor, and there was colocalization between fluorescence and bioluminescence signals. No fluorescence was observed with the control nanobody R2-800CW. CNRs for both Artemis and Pearl are 101 (SD 55) and 143 (SD 15) for 7D12-800CW, while a low fluorescence signal was detected for R2-800CW with CNRs 12 (18) and 18 (25) for Artemis and Pearl, respectively. The larger standard deviation in CNR of 7D12-800CW for the Artemis than for the Pearl could be attributed to inhomogeneous lighting conditions in the Artemis camera setup. In an open camera setting, a lower amount of excitation light tends to reach the tissue of interest, leading to excitation of a lower amount of fluorophore resulting in a lower excitation fluorescence signal that can be detected [5]. The large standard deviation is reflected in the differences in CNR of 7D12-800CW and R2-800CW between the Artemis and Pearl with *P* = 0.7 and *P* = 0.4, respectively. The CNR between 7D12-800CW and R2-800CW was statistically significantly different for the Pearl (*P* = 0.017) but not for the Artemis (*P* = 0.13). The larger standard deviation in CNR of 7D12-800CW for the Artemis could partly be attributed to inhomogeneous lighting conditions in the Artemis camera setup. Furthermore, due to short acquisition times in an intraoperative imaging setting, a smaller amount of fluorescent photons are captured per acquired frame, leading to noisier images. Lastly, the Artemis system does not have a cooled camera, increasing sensitivity to thermal noise.

#### Tumor-Specific Imaging Using 7D12-800CW, Hypopharyngeal Tumor Model

**Fig. 5:**
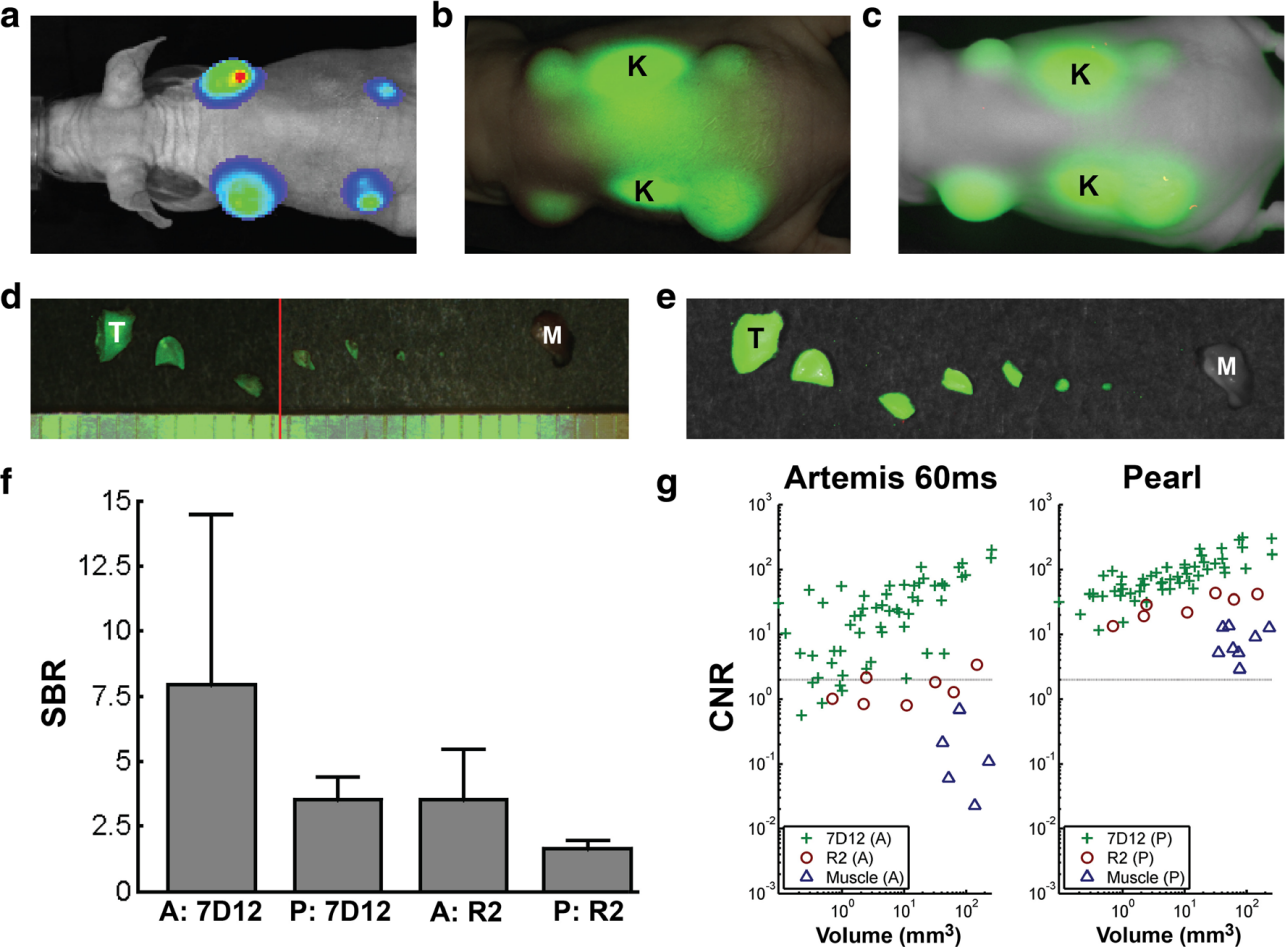
Tumor detection limit. Hypopharyngeal FaDu-luc2 tumors of different sizes visualized using bioluminescence imaged by **a** the IVIS Spectrum system could clearly be delineated *in vivo* using both **b** the Artemis and **c** Pearl. **f** *In vivo* tumor signal-to-background ratios (SBR) were calculated. Twenty-four hours after injection of 7D12-800CW and R2-800CW, all tumors were repeatedly halved until submillimeter size. Tumor pieces were measured using the **d** Artemis and **e** Pearl. **g** Contrast-to-noise ratios (*CNR*) were plotted against the volume of the tumor pieces. *Ruler lines* denote millimeters. *K* = kidney; *T* = tumor; *M* = muscle; *A* = Artemis; *P* = Pearl.

Subcutaneous FaDu-luc2 tumors could be clearly imaged with both the Artemis and Pearl camera after injection of 7D12-800CW. Histology confirmed tumor specificity of 7D12-800CW (Supplementary Fig. 1). Figure 5 shows the bioluminescent signal (a) that confirms the tumor presence and overlays from the Artemis (b) and Pearl (c). The Artemis overlay shows a high signal between the kidneys, while the Pearl overlay does not show this signal. In contrast to the Artemis, for the Pearl, the overlay can be adjusted to show that the tumors fluoresce stronger than the center of the back of the mouse where scattering increases fluorescence signal next to the kidneys. For the Artemis, the large spatial variation in illumination intensity caused a non-significant difference between SBRs for tumors with 7D12-800CW and R2-800CW (*P* = 0.09), while the difference for the Pearl was significant (*P* < 0.001) (Fig. 5f).

Figure 5d, e show an example of a halved tumor with 7D12-800CW imaged with both Artemis (d) and Pearl (e). In the Artemis overlay, part of a ruler is visible. For each tumor piece, the volume is estimated and the relation between CNR and volume is shown in Fig. 5g. Muscle tissue was included as an additional control. A clear relation between tumor volume and CNR is visible for 7D12-800CW for both Artemis and Pearl, where the Pearl in general showed a higher CNR and the Artemis a larger spread. The CNRs were significantly different for both 7D12-800CW (*P* < 0.001) and R2-800CW (*P* < 0.001). Fluorescence of tumor pieces with a CNR below 2 is practically invisible by eye (depicted by the gray horizontal line). Five tumor pieces were below this line for the Artemis, while these tumor pieces were hardly visible with the Pearl. The tumor pieces missed by the Artemis had an average size in the order ≤1 mm^3^. Although smaller pieces in general had a lower CNR, a size of 1 mm^3^ should be considered as the lower boundary, as the majority of tumor pieces of this size were visible by both cameras.

Contrast-to-noise ratios are significantly different between specific and non-specific probes for both Artemis (*P* < 0.001) and Pearl (*P* < 0.001).

### Human Liver Imaging

**Fig. 6:**
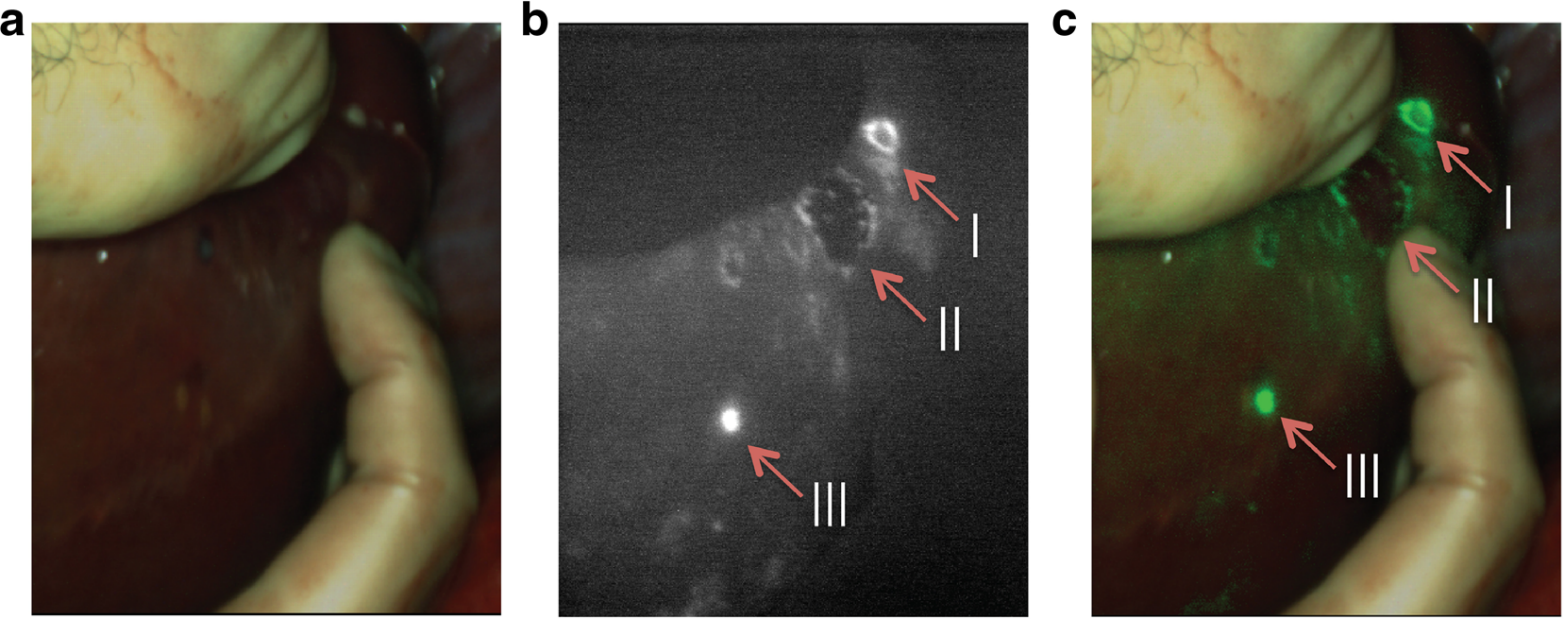
Near-infrared fluorescence imaging of colorectal liver metastases: 24 h after injection of 10 mg indocyanine green, colorectal liver metastases could clearly be identified by a rim around the tumor (*I* and *II*). Benign lesion (*III*) could be identified by fluorescence without the rim. Images are depicted in **a** visible light, **b** NIR fluorescence signal, and **c** a real-time overlay.

Three patients with liver metastases were imaged using the Artemis camera system during surgery. All three patients had metastases from colon tumors near or at the liver surface. Figure 6 illustrates the combination of images such as presented to the surgeon: visible light (Fig. 6a), NIR fluorescence signal (Fig. 6b), and a real-time overlay (Fig. 6c). The metastases in this example are recognizable due to their fluorescent rim. Benign lesions could be differentiated from malignant lesions by a lack of this fluorescent rim around the tumor, as was confirmed by pathologic analysis [20]. A total of six lesions with fluorescent rim were identified during surgery with NIR fluorescence, and all showed to be malignant after pathologic evaluation. No false-negative nodules were found. One lesion in segment 6 of the liver was initially missed by eye but was clearly visualized using the Artemis system.

## Discussion

Fluorescence-guided surgery is a high potential imaging technique that provides surgeons with real-time information about vital structures, tumor margins, and regional disseminated disease. By real-time feedback of tumor margins and vital structures, tumors could be radically resected while healthy tissue can be preserved. A dedicated NIR fluorescence camera is vital as NIR light is invisible to the human eye. In this study, we evaluated the Artemis NIR fluorescence camera system. Its performance in detecting ICG and IRDye 800CW was assessed and was put in perspective by comparison with the preclinical Pearl imaging system. Furthermore, we demonstrated its utility in detecting and guiding resection of cervical SLNs using ICG, as well as primary tongue tumors and hypopharyngeal tumors using an EGFR-targeting nanobody conjugated to IRDye 800CW. Next, first in-human clinical data using the Artemis was shown by the detection of liver metastases using ICG.

The efficacy of NIRF camera systems and the final real-time fluorescence imaging results are dependent on multiple factors. These factors include the interplay between the type of probe or fluorophore used, probe concentration injected, concentration of probe at the location of interest, tumor size, optical properties, and the camera system.

The Artemis was evaluated for the use in two frequently used FGS procedures because there is a major difference in dye and in concentration of dye at the site of interest between both procedures. The near-tumoral injected ICG in SLN mapping is highly concentrated compared to the intravenously injected conjugate of IRDye 800CW and a targeting moiety in tumor-specific imaging. Furthermore, ICG and IRDye 800CW differ in excitation spectra, emission spectra, and quantum yield. In general, imaging results are more adequate when a fluorophore has a high quantum yield and there is a high concentration at the site of interest.

The second variable that intervenes with the efficacy of a NIRF camera is the injected concentration of probes. For the experiments *in vivo*, the optimal concentration was chosen. The optimal concentration of ICG was extrapolated from the ICG dilution series *in vitro*. In the choice of the injected dose *in vivo* (100 μM), dilution of ICG after intratumoral injection was taken into consideration. Concentrations of 7D12-800CW (50 μM) and R2-800CW (μM) were chosen from earlier studies [14].

Next, the size of the tumor determines the imaging results. We assessed the detection limit of a FaDu-luc2 tumor nodule size that could be detected using the nanobody 7D12-800CW. For that, tumor pieces were subdivided into halves until submillimeter size. Since 7D12-800CW is homogenously distributed throughout the tumor [13, 14] (Supplementary Fig. 1), subdivision was justified. A size of 1 mm^3^ could be considered as the lower boundary of detection, as the majority of tumor pieces of this size were visible. A tumor size of 1 mm^3^ is considered to contain around three million tumor cells. Using this setup, lower amount of cells that could be clinically significant would not be detected. Obviously, a detection limit is dependent on multiple factors like the probe that is used, the observer, the concentration and pharmacokinetics of the probe, the target that is chosen, and the amount of receptors in the tissue of interest. Furthermore, *in vivo*, the optical properties of the tissue of interest and overlaying tissue determine in a great extent what size of tumor tissue can be detected. Again, results were compared to the Pearl to validate the Artemis data.

Despite successful utility of the Artemis in our preclinical experiments and the successful utility of the Artemis in a clinical setting, several improvements can be made to increase the applicability and imaging reliability. First, the illumination intensity as projected by the Artemis sharply decreases near the edge of the imaged field. This causes the apparent fluorescence intensity to have a strong dependence on the location in the image. In the experiment using cell lines *in vitro*, we showed that both the Artemis and Pearl were able to image the same relationship between number of cells and intensity for the tumor-specific 7D12-800CW probe. For the non-specific R2-800CW, both cameras showed a flat profile. The Artemis signal was less consistent than that of the Pearl for low cell counts with 7D12-800CW and for the cells with R2-800CW. This can be attributed to a less inhomogeneous illumination of the sample than for the Pearl. Precise positioning of the sample when comparing fluorescence intensities is thus essential for the Artemis while less critical for the Pearl. This spatial illumination variation is also visible in Fig. 5f, as a large standard deviation on the SBRs for the *in vivo* tumors resulting in a non-significant difference between SBRs for tumors with 7D12-800CW and R2-800CW (*P* = 0.09).

Second, a possible improvement lies in filtering the excitation light. The filters that are intended to block the excitation light do not block most specular reflections. If available, filters with a higher optical density would solve this problem or cross-polarization could be used. Results of the impairment in blocking reflections can be seen in the baseline experiment for evaluating the sensitivity of the Artemis system that consisted of measuring the signal from fluorophores in concentration series. For the real-time setting of 60 ms exposure time per image, the Artemis was able to measure concentrations of both ICG and 800CW of 10^−2^ μM and higher. The control Pearl camera showed that fluorophores were present in wells with lower concentrations. This lower boundary was not solely due to sensitivity limits of the Artemis camera but also due to reflected excitation light that was not sufficiently filtered out. For concentrations smaller than 10^−2^ μM, these reflections were stronger than the fluorescence signal.

Third, the current camera has a rather low depth of field. Since the camera does not have an autofocus mechanism, this requires frequent adjustment of the focus. An additional problem with the current Artemis camera systems is a focal length difference between the visible light and near-infrared channels. This requires changing the focus between a sharp visible light and near-infrared fluorescence image at close imaging distances (<15 cm).

A last possible improvement is the dynamic range of the camera. This lack of range is visible in the images with concentration ranges; only a few concentrations are between the lower detection boundary and the saturation boundary. Although such a wide variation in concentrations is not to be expected in clinical applications and overexposure is not a big issue, large variations in working distance during surgery also lead to large intensity variations of the fluorescence signal. The visible light channel also tended to be overexposed, even at the least sensitive camera settings.

## Conclusion

NIR fluorescence-guided surgery could aid surgeons in real-time visualization of tumors, SLNs, and vital structures to ensure a radical resection, adequate staging, and minimize damage to normal tissue. In this study, we evaluated the Artemis system and assessed the minimal detection limit of tumor-specific imaging using an EGFR-targeting nanobody. Furthermore, we demonstrated the possibility of fluorescence-guided resection of head and neck tumors and sentinel lymph nodes. At last, we demonstrated the use of the Artemis system for the detection and fluorescence-guided resection of liver metastases in a first in-human clinical trial. Based on this study, although improvements can be made, we think the Artemis system has demonstrated its utility in fluorescence-guided cancer surgery.

## Electronic Supplementary Material

Below is the link to the electronic supplementary material.

Supplementary Fig. 1 Histology of subcutaneous human hypopharyngeal squamous cell carcinomas: fluorescence of 7D12-800CW is observed in the tumor, implicating tumor specificity of 7D12-800CW. Shown are hematoxylin and eosin (HE) stainings, near-infrared (NIR) fluorescence images of 7D12-800CW and R2-800CW and anti-human wide spectrum cytokeratin stainings indicating the presence of tumor cells. (PDF 766 kb)

## References

[1] Keereweer S, Mol IM, Vahrmeijer AL (2012). Dual wavelength tumor targeting for detection of hypopharyngeal cancer using near-infrared optical imaging in an animal model. Intl J Cancer.

[2] Vahrmeijer AL, Hutteman M, Van Der Vorst JR (2013). Image-guided cancer surgery using near-infrared fluorescence. Nat Rev Clin Oncol.

[3] Frangioni JV (2008). New technologies for human cancer imaging. J Clin Oncol.

[4] Adams KE, Ke SK (2007). Comparison of visible and near-infrared wavelength-excitable fluorescent dyes for molecular imaging of cancer. J Biomed Opt.

[5] Keereweer S, Van Driel PB, Snoeks TJ (2013). Optical image-guided cancer surgery: challenges and limitations. Clin Cancer Res.

[6] Gioux S, Choi HS, Frangioni JV (2010). Image-guided surgery using invisible near-infrared light: fundamentals of clinical translation. Mol Imaging.

[7] Schaafsma BE, Mieog JS, Hutteman M (2011). The clinical use of indocyanine green as a near-infrared fluorescent contrast agent for image-guided oncologic surgery. J Surg Oncol.

[8] Mcmahon J, O’Brien CJ, Pathak I (2003). Influence of condition of surgical margins on local recurrence and disease-specific survival in oral and oropharyngeal cancer. Brit J Oral Maxillofac Surg.

[9] Huston TL, Simmons RM (2005). Locally recurrent breast cancer after conservation therapy. Am J Surg.

[10] Meric F, Mirza NQ, Vlastos G (2003). Positive surgical margins and ipsilateral breast tumor recurrence predict disease-specific survival after breast-conserving therapy. Cancer.

[11] Rusthoven KE, Raben D, Song JI (2010). Survival and patterns of relapse in patients with oral tongue cancer. J Oral Maxillofac Surg.

[12] Schulze T, Bembenek A, Schlag PM (2004). Sentinel lymph node biopsy progress in surgical treatment of cancer. Arch Surg.

[13] Oliveira S, Van Dongen GA, Stigter-Van Walsum M (2012). Rapid visualization of human tumor xenografts through optical imaging with a near-infrared fluorescent anti-epidermal growth factor receptor nanobody. Mol Imaging.

[14] Van Driel PB, Van Der Vorst JR, Verbeek FP et al (2013) Intraoperative fluorescence delineation of head and neck cancer with a fluorescent anti-epidermal growth factor receptor nanobody. Intl J Canc 10.1002/ijc.28601 PMC3960332 24222574

[15] Dolk E, Van Vliet C, Perez JM (2005). Induced refolding of a temperature denatured llama heavy-chain antibody fragment by its antigen. Proteins.

[16] Gainkam LO, Huang L, Caveliers V (2008). Comparison of the biodistribution and tumor targeting of two 99mTc-labeled anti-EGFR nanobodies in mice, using pinhole SPECT/micro-CT. J Nucl Med: Off Publ Soc Nucl Med.

[17] Roovers RC, Laeremans T, Huang L (2007). Efficient inhibition of EGFR signaling and of tumour growth by antagonistic anti-EFGR nanobodies. Cancer Immunol Immunother: CII.

[18] Mieog JS, Vahrmeijer AL, Hutteman M (2010). Novel intraoperative near-infrared fluorescence camera system for optical image-guided cancer surgery. Mol Imaging.

[19] Tomayko MM, Reynolds CP (1989). Determination of subcutaneous tumor size in athymic (nude) mice. Cancer Chemother Pharmacol.

[20] Van Der Vorst JR, Schaafsma BE, Hutteman M (2013). Near-infrared fluorescence-guided resection of colorectal liver metastases. Cancer.

