# Supplementary material for: Characterization and Evaluation of the Artemis Camera for Fluorescence-Guided Cancer Surgery
Source: Mol Imaging Biol. 2014 Oct 25;17(3):413–23. doi: 10.1007/s11307-014-0799-z (PMC4422838; doi:10.1007/s11307-014-0799-z)
Supplement: Supplementary file 1 — Histology of subcutaneous human hypopharyngeal squamous cell carcinomas: fluorescence of 7D12-800CW is observed in the tumor, implicating tumor specificity of 7D12-800CW. Shown are hematoxylin and eosin (HE) stainings, near-infrared (NIR) fluorescence images of 7D12-800CW and R2-800CW and anti-human wide spectrum cytokeratin stainings indicating the presence of tumor cells. (PDF 766 kb) [file 11307_2014_799_MOESM1_ESM.pdf]

Supplemental Information

Characterization and Evaluation of the Artemis Camera for fluorescence-guided Cancer Surgery

Molecular Imaging and Biology

P.B.A.A. van Driel[1*], M. van de Giessen[2*], M.C. Boonstra[3], T.J.A. Snoeks[1], S. Keereweer[4], S. Oliveira[5,6], C.J.H. van de Velde[3], B.P.F. Lelieveldt[2], A.L. Vahrmeijer[3], C.W.G.M. Löwik[1], J. Dijkstra[2]

[*] Both authors contributed equally to this work and share first-authorship.

Leiden University Medical Center, Leiden, The Netherlands, departments of Radiology, Molecular imaging[1] and image processing[2] and Surgery[3]

Erasmus Medical Center, Rotterdam, The Netherlands, Department of Otorhinolaryngology & Head and Neck Surgery[4]

Utrecht University, Utrecht, The Netherlands, Department of Biology[5]

University Medical Center Utrecht, Utrecht, The Netherlands, Department Pathology[6]

Corresponding author: P.B.A.A. van Driel, Leiden University Medical Center, P.O. Box 9600, 2300 RC Leiden, The Netherlands, tel: +31715263075

Characterization and Evaluation of the Artemis Camera for fluorescence-guided Cancer Surgery – supplementary figure

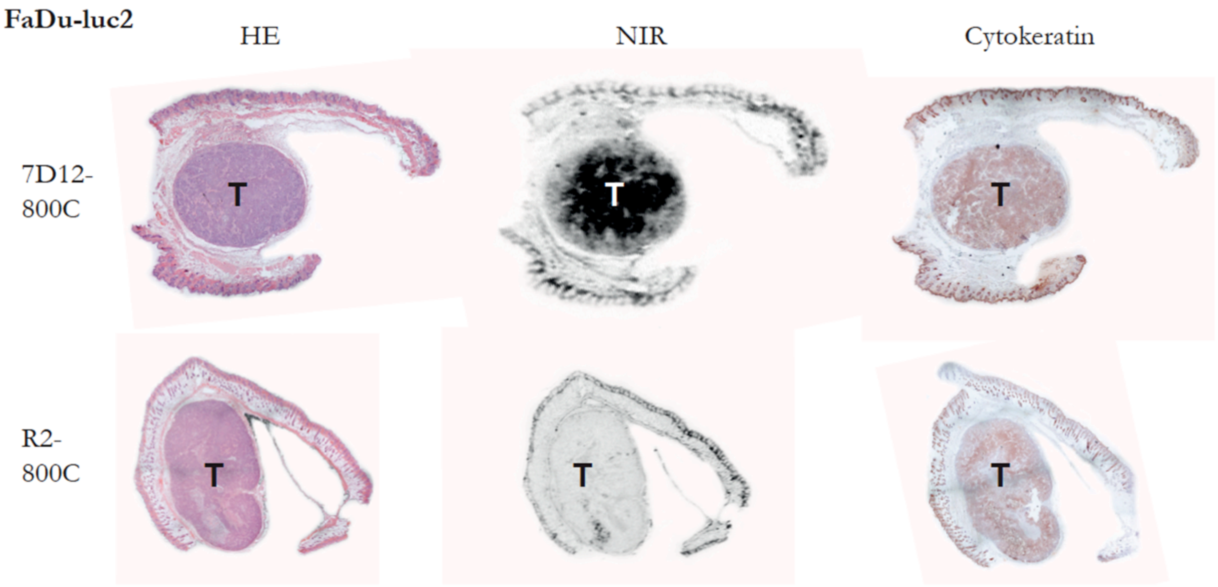

Supplementary figure I. Histology of subcutaneous human hypopharyngeal squamous cell carcinomas: fluorescence of 7D12-800CW is observed in the tumor, implicating tumor specificity of 7D12-800CW. Shown are hematoxylin and eosin (HE) stainings, near-infrared (NIR) fluorescence images of 7D12-800CW and R2-800CW and anti-human wide spectrum cytokeratin stainings indicating the presence of tumor cells.
